# Research ethics consultation: an attempt and 5-year experience in a Japanese University Hospital

**DOI:** 10.1186/s13104-018-3772-0

**Published:** 2018-09-12

**Authors:** Hiroaki Yanagawa, Rumi Katashima, Chiho Sato, Kenshi Takechi, Hiroshi Nokihara, Chikako Kane, Masayuki Chuma, Yuki Aoe

**Affiliations:** Clinical Trial Center for Developmental Therapeutics, Tokushima University Hospital, Kuramoto-cho 2, Tokushima, 770-8503 Japan

**Keywords:** Research ethics consultation, Research ethics, Clinical research, Japan, Regulation compatibility

## Abstract

**Objective:**

Research ethics consultation is an advisory activity that differs from ethics committees, and its role is not yet widely known in Japan. Research ethics consultations were started in 2012 by members of the Clinical Trial Center of Tokushima University Hospital, a support section for clinical trials. We analyzed the research ethics consultation records from Tokushima University Hospital during the 5-year period of 2012–2016 to examine the Japanese context of research ethics consultation.

**Results:**

During the study period, 125 research ethics consultations were carried out, 115 (91%) before starting studies. All but one request were from investigators at Tokushima University. The main issue was compatibility with guidance and regulations (n = 74, 67.2%), such as ethical handling of human biological specimens and information utilized in research; only 6 (4.8%) requests involved research ethics issues that investigators face in their research. Therefore, it is necessary to expand the consultation function, with a nationwide system of consultant education and data sharing. Moreover, standardization of consultation should be considered.

## Introduction

Attention to research ethics is a key element of biomedical research, and the role of research ethics committees is well established in many countries, including Japan [1]. Research ethics consultation is a different activity from ethics committees; defined as an advisory activity available throughout the life cycle of a study, it involves interactions between researchers or other stakeholders in the research enterprise and one or more individuals knowledgeable about the ethical considerations of research regarding any aspect of planning, conducting, interpreting, or disseminating the results of research related to human health and well-being [2]. Research ethics consultation has been reported from institutes in the United States, including Stanford University [3, 4], Johns Hopkins Bloomberg School of Public Health [5, 6], and Weill Cornell Medical College [7]. Research ethics consultation was promoted by the Clinical and Translational Science Award (CTSA) Program initiated by the National Center for Research Resources in 2005, as the request for applications noted the importance of an institutional commitment to supporting ethics [8]. In response to this movement, many institutions in the United States developed research ethics consultation services.

In Japanese clinical research, the infrastructure for clinical trials for drug approval, also called registration trials, proceeded after the introduction of the good clinical practice (GCP) guidelines originally approved by the International Conference on Harmonisation of Technical Requirements for Registration of Pharmaceuticals for Human Use in 1998. To provide infrastructure for registration trials and to comply with the GCP guidelines, organization and personnel training were pushed forward. However, in 2001–2003, ethics guidelines that apply to clinical studies other than registration trials were established by the government ministries [9], demanding increased consultation enforcement. Thus, the talented person in conjunction with the registration trials handles the consultation with a researcher, including research ethics in many institutions. At Tokushima University Hospital, an academic hospital in a rural area of Japan, we started to handle consultations, mainly for issues concerning research ethics, by securing time in 2012. Here, we report 5-years of experience with our consultation service.

## Main text

### Methods

#### Research ethics consultation at Tokushima University Hospital

To provide infrastructure for registration trials and to comply with the GCP guidelines at Tokushima University Hospital, the Clinical Trial Center for Developmental Therapeutics (CTCDT) was established in April 1999. The original aim of the CTCDT was to manage and promote registration trials by liaising with clinical research coordinators (CRCs) [10]. After the enforcement of government ethics guidelines, which apply to clinical studies other than registration trials, the CTCDT was set-up to meet the requirements of the various ethics guidelines. In July 2002, the Academic Office of the Ethics Committee was set-up as part of the CTCDT. One medical doctor and two scientists other than the ethics committee members work with persons in the administrative division. The office started advising investigators and conducting document pre-checks. Based on the experience with these activities, these three members started work in research ethics consultation in April 2012. Essentially, inquiries relating to simple procedure and application documents are not included in our research ethics consultation. Consultation by the ethics committee is not included in our research ethics consultation, as this activity is provided on other occasions.

#### Analysis of consultation records

We have been recording our research ethics consultations, including the following elements: client information, question, assessment and analysis, and recommendation. In the present study, we retrospectively analyzed these records. In a previous report of research ethics consultations from a Japanese institute, Kamisato et al. [11] divided their research ethics consultations into four categories: consultations on research ethics issues that investigators face in their research, consultations on compatibility with guidance and regulations, consultations on the application of ethics reviews (except documents themselves), and consultations on application documents for the ethics committee. We essentially categorized our consultations based on this report, but consultations in the last category (application documents for the ethics committee) were not included in our analysis. Instead, we added a category of other inquiries that did not fit into the other three categories. No statistical analysis was applied.

### Results

#### Annual numbers and timing of research ethics consultations

The number and timing of research ethics consultations are shown in Table 1. A total of 125 research ethics consultations were performed during the study period at Tokushima University Hospital, and more than 90% of these consultations occurred before starting studies. All requests except one were from investigators at Tokushima University. The exceptional case was from the administrative office regarding the ethical review system at Tokushima University.

**Table 1:** Annual numbers and timing of research ethics consultations at Tokushima University Hospital

	Before starting studies (%)	After starting studies (%)	Total
2012	34 (97%)	1 (3%)	35
2013	17 (89%)	2 (11%)	19
2014	24 (100%)	0 (0%)	24
2015	16 (84%)	3 (16%)	19
2016	24 (86%)	4 (14%)	28
Total	115 (91%)	10 (9%)	125

#### Issues raised in research ethics consultations

As shown in Table 2, 84 (67.2%) were consultations on compatibility with guidance and regulations (Category B). Among 84 consultations in Category B, the detailed topics are shown in Table 3. As shown, ethical handling of information utilized in research was most frequently consulted topic. Fewer consultations (n = 6) were on research ethics issues that investigators face in their research (Category A). Among these, the main topic was non-invasive prenatal genetic testing (n = 5) and another topic was development of medical device. Category C (application of ethics review) included 16 consultations of new application, 7 consultations of application of protocol alterations, and 4 consultations of ethics review system of Tokushima University. Category D (others) included 4 consultations of scientific aspects of clinical research, such as design of clinical trials, data and safety monitoring board and monitoring, and other 4 consultations were issues of clinical practice rather than clinical research. No consultation was on a new type of modality as a result of translational research (data not shown).

**Table 2:** Issues of research ethics consultations in 5 years at Tokushima University Hospital

Category	Issues	2012	2013	2014	2015	2016	Total (%)
A	Issues on research ethics that investigators face in their research	2	0	0	0	4	6 (4.8%)
B	Compatibility with Japanese government guidelines	20	7	14	14	12	67 (53.6%)
	Compatibility with other guidance and regulations	3	4	6	2	2	17 (13.6%)
C	Application of ethics review (except documents themselves)	7	6	4	2	8	27 (21.6%)
D	Others	3	2	0	1	2	8 (6.4%)
Total		35	19	24	19	28	125

**Table 3:** Topics of research ethics consultations of compatibility with Japanese government guidelines and other guidance and regulations

Topics	2012	2013	2014	2015	2016	Total (%)
Ethical handling of human biological specimens	2	2	1	5	3	13 (15.5%)
Ethical handling of information utilized in research	6	2	3	6	0	17 (20.1%)
Necessity and way of informed consent	2	0	5	1	0	8 (9.5%)
Categorization of invasiveness	0	0	0	1	1	2 (2.4%)
Categorization of intervention	6	0	3	1	3	13 (15.5%)
Application of ethical guidelines	4	3	2	0	5	14 (16.7%)
Examination of functions of food and supplements	3	0	0	1	2	6 (7.1%)
Advanced medicine	0	2	1	1	0	4 (4.8%)
Trials for governmental approval (registration trials)	0	1	2	0	0	3 (3.6%)
Gene therapy	0	1	0	0	0	1 (1.2%)
Regenerative medicine	0	0	1	0	0	1 (1.2%)
Good clinical practice	0	0	1	0	0	1 (1.2%)
Insurance in clinical trials	0	0	1	0	0	1 (1.2%)
Total	23	11	20	16	14	84

### Discussion

In biomedical research, research ethics is undoubtedly an important element. Apart from an ethical review by an ethics committee, research ethics consultation services have started to cope with issues in research ethics. As defined by Bescow et al. [2], the provider of a research ethics consultation service is considered to be knowledgeable about the ethical considerations in research. Sharp et al. [12] concluded that a research ethics consultation service should be provided by individuals with relevant content expertise derived from course work or personal experience, and teaching research ethics to advanced students and prior scholarship on topics in research ethics are good indicators of such expertise.

In Japan, few courses at the university are specialized in research ethics, but there are still a few faculty members of research ethics. The research ethics consultation providers at Tokushima University Hospital consist of one physician (the Director of the CTDCT) and two scientists in the clinical trial support section. This is in line with previous reports [13, 14]. Kamisato et al. [13] reported the results of a survey of participants in a meeting organized with persons who respond to inquiries about research ethics at their facilities. Among 20 facilities, the bioethics faculty, clinical trial support section members, and administrative office members mainly responded to these inquiries in 8 facilities, 6 facilities, and 6 facilities, respectively. Aizawa et al. [14] reported the results of a survey of participants regarding the training opportunities in research ethics that they provided; among 119 participants (54 ethics committee members, 32 CRCs, 27 administrative office members, 23 investigators, 6 ethicists, and 9 others, multiple answers allowed), only 49 (41.2%) had specific departments that respond to inquiries about research ethics at their facilities. Among these departments, clinical trial support section members and administrative office members mainly responded to inquiries in 28 facilities (40.6%) and 19 facilities (27.5%), respectively. Moreover, 55 (46.2%) had not or were not aware of the presence of research ethics specialists at their facilities. We have to consider that these previous reports were surveys of facilities that were able to dispatch members in charge of research ethics for these meetings and/or training opportunities, and the situation could be poorer in ordinary research institutions.

Our research ethics consultation providers were not ethics committee members. Ethics committee members may serve in research ethics consultation at some facilities, but we consider it desirable to build a system in which research ethics consultation acts independently from the ethics committee, clarifying the difference between advice and review. Currently, our research ethics consultation providers support the ethics committee with an annual survey of the ongoing state of approved studies, the maintenance of a web-based application system, and pre-checking application documents. However, separating practical support of the ethics committee from research ethics consultation could be desirable.

The role of research ethics consultation is to advise on research ethics beyond simple guidance on compatibility, and this point makes research ethics consultation another activity rather than a simple response to inquiries from investigators. However, the situation is different in Japan. In Kamisato et al.’s [10] analysis of their 234 research ethics consultations in 2009, only 3 (1.3%) requests were consultations on research ethics issues that investigators face in their research. Although we started our research ethics consultation independently from our ordinary activity to respond to general inquiries from investigators, the main issue was consultation on the compatibility with guidance and regulations (67.2%), and only 6 (4.8%) requests were consultations on research ethics issues that investigators face in their research (Table 2), which is in accordance with the previous study by Kamisato et al. [11].

In research ethics consultation, it is necessary to expand the consultation function to research ethics issues that investigators face in their research; therefore, the quality of research ethics consultation providers is important. Under the present conditions, exchanging experiences among research ethics providers may be a method to consider. Moreover, it is necessary to have a nationwide system for advancing research ethics consultants, as well as facilities securing an employment system for these experts. In the CTSA consortium, a consultation group organizes and shares data on research ethics consultation, working towards the standardization of research ethics consultation [15]. Porter et al. [16] recently reported that the national Research Ethics Consultation Collaborate collected more than 350 research ethics consultations in a repository and published 18 challenging cases with accompanying ethical commentaries. In an attempt at data sharing regarding research ethics consultation in Japan, the Office for Research Ethics and Bioethics of National Cerebral and Cardiovascular Center has website that accepts nationwide research ethics consultations to share cases and recommendations [17]. We have to expand this activity and standardize the aims, policies, and operation procedures in the future.

### Conclusion

We analyzed 125 research ethics consultations at Tokushima University Hospital and found that the main issue was consultation on the compatibility with guidance and regulations. It is necessary to expand the consultation function to the issues on research ethics that investigators face in their research. Establishment of a nationwide education system of consultants and data sharing with standardization of consultation is warranted.

## Limitations

Several limitations should be considered in the present study. The present study was conducted in one university hospital in Japan. Although previous reports described similar situation in handling inquiry about issues in research ethics from investigators [11, 13, 14], the analysis does not wholly reflect the status of research ethics consultation in Japan. Moreover, the research infrastructure varies among various countries, generalizability of the results in the present study in international settings should be examined in future studies.

## Abbreviations

CTSA: Clinical and Translational Science Award
GCP: good clinical practice
CTCDT: Clinical Trial Center for Developmental Therapeutics
CRC(s): clinical research coordinator(s)
